# Exploring the contextual factors that impact the dementia family caregiving experience in Soweto township, South Africa

**DOI:** 10.1177/14713012221117905

**Published:** 2022-08-08

**Authors:** Aqeela Mahomed, Chrisma Pretorius

**Affiliations:** Department of Psychology, 26697 Stellenbosch University, Stellenbosch, South Africa

**Keywords:** township, dementia, family caregiver, financial burden, psychosocial stressors

## Abstract

Townships and rural areas endure difficult circumstances such as poverty, unemployment, low educational levels, unstable income sources, socioeconomic deprivation and the lack of transportation. Furthermore, psychosocial issues such as crime, violence and substance abuse are additional contextual factors prevalent within South African townships. There has been a paucity of research focussing on the impact of contextual and socioeconomic conditions on the dementia family caregiver experience. This qualitative study aimed to address this gap. Semi-structured interviews were conducted with 30 family caregivers via purposeful sampling methods. Data analysis using Reflexive Thematic Analysis (RTA) generated four broad themes, namely – (*1). Poverty, (2). Crime, Violence and Substance Abuse, (3). Practical Challenges and (4). A Sense of Normalcy.* The findings of this study depict the socioeconomic conditions of family caregivers living in Soweto and its impact on dementia caregiving. The majority of the family caregivers in this study were unemployed and identified the financial aspects of caregiving as a significant strain. Beyond financial aspects, practical challenges that some family caregivers reported included spatial constraints and insufficient material resources. Caregivers raised safety concerns due to the dangers that this socioeconomic context posed. However, there was an implied sense of normalcy and a reluctance to identify challenges that caregivers endured. Recommendations for further research and its implications for public health policies and important initiatives to advocate for dementia caregivers and their family members are outlined.

## Introduction

It has been well-established that the prevalence of dementia has developed into one of the most pressing health concerns, especially among lower–middle–income countries (LMICs) (De Jager et al., 2017; Kehoua et al., 2019; Owokuhaisa et al., 2020). In Sub-Saharan Africa (SSA), prevalence data on dementia is severely lacking (Mubangizi et al., 2020) despite estimates of prevalence rates increasing dramatically as the population ages (Kehoua et al., 2019). The same concern was highlighted by South African researchers in 2015, where De Jager and colleagues delineated ‘a pressing need’ (p. 189) for studies to be conducted to estimate the public health concern among the South African population. This is important as the responsibility of taking care of individuals with dementia is assumed by family caregivers, who are ill-equipped to do so – leading to caregiver burden (Lindeza et al., 2020).

Research by Joubert (2005) reported that the largest prevalence rates of family caregivers are amongst the Black South African population – “double the rate” (p. 107) relative to the other designated population groups^1^ in South Africa. This discrepancy among population groups involves differences regarding the strain of disease and disability, living arrangements, access to formal healthcare services and cultural approaches to frail care (Joubert, 2005). Geographically, the prevalence of family caregivers was reported to be significantly higher in rural areas due to the lack of availability of institutional services and South Africa’s policy on de-institutionalisation and community-based care (De Jager et al., 2015; Kalula & Petros, 2011; Mkhonto & Hanssen, 2018) – thereby compounding the abovementioned factors (Joubert, 2005).

Furthermore, the prevalence of caregiving was strongly correlated with education, employment status and monthly income. In contrast, lower educational and income levels and unemployment was associated with a higher prevalence of caregiving (Joubert, 2005). The financial costs of caregiving are considered in monetary value and the perceived financial implications of dementia caregiving (Lai, 2012). The daily expenses of taking care of an individual with dementia include transportation, medication, medical supplies, caregiving equipment and household necessities (Hollander et al., 2009; Lai, 2012). However, the perceived financial implications refer to the sacrifices made by the caregiver due to their caregiving responsibility. As such, family caregivers who are employed require more time off work, are subject to more interruptions at work and may even miss career opportunities or leave work due to the demands of their caregiving responsibilities (Aoun et al., 2005; Lai, 2012; Schulz et al., 2003). Findings of a South African study conducted with spousal caregivers of their partners with Dementia of the Alzheimer’s type (DAT) indicated that caregivers not only gave up their work, but their levels of productivity and time spent at work decreased due to their caregiver obligations (Valoo, 2016). Furthermore, spousal caregivers in this study highlighted financial problems due to medical costs and supplies for their partners with DAT (Valoo, 2016).

Although costs of dementia caregiving are high and the financial burden becomes that of the family caregiver, no remuneration is provided to mitigate this strain, especially in LMICs (Kehoua et al., 2019). South African studies have highlighted this (Bosch, 2014; Gurayah, 2015) and challenges that may arise from rural and low-income communities such as townships that already endure difficult living conditions. Townships in South Africa refer to neighbourhoods delineated under the constructs of colonialism and apartheid to provide residences for the labour force from economic centers in the city (Scheba & Turok, 2019). It has been posited that townships and rural areas may be subjected to worse consequences (De Jager et al., 2015; Gurayah, 2015) due to factors such as poverty, unemployment, low educational levels, unstable income sources, socioeconomic deprivation and the lack of transportation (Kalula & Petros, 2011; Visser & Moleko, 2012). Furthermore, psychosocial issues such as crime, violence and substance abuse are additional contextual factors prevalent within South African townships (Motseke, 2013; Sedibe & Hendricks, 2021). Despite these concerns, there has been a paucity of research with a specific focus on the impact of contextual and socioeconomic conditions on the dementia family caregiver experience. This study aims to address this gap by exploring the contextual factors that may impact the dementia family caregiving experience in Soweto – the largest Black township in South Africa (Statistics South Africa [SA], 2018).

## Methods

### Research context

South Western Township in Johannesburg, known as Soweto, is home to over 1 million people – 98.5% of whom are Black African (Census, 2011; Statistics SA, 2018). Notably, researchers estimate a much larger population of 3.5 million and emphasize the difficulty of obtaining precise statistics due to the scores of illegal immigrants living in Soweto’s informal settlements (Briscoe, 2002; Creighton, 2003; Ramchander, 2004). The establishment of informal settlements occurred after the pass laws – which restricted the movement of Black people in South Africa under the Apartheid regime – were abolished (Ian, 1999). People who lived in rural areas migrated to Soweto and built shacks on vacant land, which have persisted and grown to date – despite subsidized housing since constitutional democracy was obtained in South Africa in 1994 (Mears, 2011). The Reconstruction and Development Programme (RDP), instituted by the first democratic government (African National Congress; ANC), aimed to redress the inequalities created by the apartheid regime in disadvantaged townships by providing housing and access to essential services, amenities and develop infrastructure (Moolla et al., 2011). The provision of water, electricity, basic sanitation and the building of roads, schools and healthcare facilities was among the priority areas identified by the ANC for Soweto (Moolla et al., 2011).

While there have been vast improvements in Soweto’s infrastructure and community facilities through various projects (see Sibiya, 2012), there are many suburbs in Soweto that are riddled with service delivery challenges, poor housing quality, spatial restrictions leading to overcrowding and inaccessibility to healthcare facilities (Harvey, 2007; Mabitsela, 2012; Moolla et al., 2011; Morontse, 2010; Wafer, 2012). This seems to be the case, even for better-developed suburbs like Protea Glen – where residents fall in the average working class and qualify for home loans (Kemp & Vyas-Doorgapersad, 2020; Sibiya, 2012). Kemp and Vyas-Doorgapersad (2020) particularly identified poor sanitation, hygiene and road conditions as problematic and highlighted illegal electricity connections due to high electricity costs and poor maintenance of electricity infrastructure. As a result of the poor living conditions that persist, service delivery protests have become a regular occurrence in Soweto (Wafer, 2012).

Regarding public health facilities, Soweto has 23 primary healthcare (PHC) day clinics and two public hospitals, namely, Chris Hani Baragwanath Academic Hospital (CHBAH) and Jabulani Hospital, which operate 24 h daily (Adedini et al., 2020). Even though PHCs are located within a 2-km radius of most residences in various suburbs (Adedini et al., 2020), challenges such as long queues, inadequate service provision and lack of medical treatment have been identified by primary healthcare users (Gwabeni, 2016; Mabitsela, 2012; Morontse, 2010). Furthermore, Gwabeni (2016) asserted that these challenges were accepted and treated as ‘normal’ (p. 51) for the younger to middle-aged population but posed a ‘major issue’ (p.52) for the elderly. Specifically, the elderly struggle with long waiting times (Kelly et al., 2019), cold early mornings (Gwabeni, 2016; Kelly et al., 2019; Morontse, 2010) and ‘rude’/‘disrespectful’ (Gwabeni, 2016, p. 52) treatment by nurses. Transportation to the clinics is also problematic due to financial constraints if individuals are too ill to walk to clinics or, in the case of the elderly, have limited mobility/functional capacity (Gwabeni, 2016; Kelly et al., 2019). In some suburbs, there are no clinics at all (Harvey, 2007; Kotane, 2016), which poses significant barriers to accessible healthcare.

According to a City of Johannesburg (CoJ) (2018) profile analysis report, 43% of Soweto’s residents are unemployed. High unemployment rates have given rise to substance abuse, violence and criminal activity (Burton, 2003; Ramchander, 2004). The absence of Police stations in some suburbs has also been a contributing factor (Harvey, 2007; Kotane, 2016). Despite the abovementioned challenges that perpetuate poverty and social and economic deprivation, Soweto is gradually evolving through urban renewal projects and remains an international symbol of political freedom (Ramchander, 2004; Sibiya, 2012).

In terms of socio-cultural aspects, residents of Soweto comprise various indigenous groups who converse in nine African languages, namely, isiXhosa, Tshivenda, Setswana, Sepedi, isiZulu, Sesotho, isiNdebele and Shangaan (Mabogane & Callaghan, 2002; Ramchander, 2004). Furthermore, according to Ramchander (2004), ‘more than 80% of the population in Soweto speak English’ (p. 30). Although present-day culture in Soweto appears to have both ethnic and Western influences (Mabogane & Callaghan, 2002), a significant subset of the population is grounded in African culture and tradition (Ramchander, 2004). Another prominent feature in Soweto is the ‘Spaza’ shops, commonly built-in residents’ garages, outdoor rooms or areas near public transportation hubs to provide basic food and clothing essentials within the community (Moolla et al., 2011; Ramchander, 2004). In terms of transportation, minibus taxis, buses and trains are Soweto’s predominant modes of transport. Lastly, everyday recreational activities in Soweto include beer drinking and soccer – of which there are approximately 2500 shebeens, 220 taverns, 120 soccer fields and four stadiums (Creighton, 2003; Ramchander, 2004).

### Participants and recruitment

Ethical clearance was granted for this study by the Research Ethics Committee at Stellenbosch University (PSY-2019-10582). Researchers of this study collaborated with *Alzheimer’s South Africa* – a Non-Governmental Organization (NGO), to identify 30 family caregivers via purposive sampling techniques who met the inclusion criteria for the study (see Table 1). Inclusion criteria were shared with the *Alzheimer’s South Africa* Soweto office coordinator, who established initial contact with suitable participants to introduce the research objectives and explain the interview process.

**Table 1.** Inclusion Criteria for the study.

1. Caregivers should have already received a diagnosis of dementia for their family member
2. Caregivers should be a family member of the person with dementia
3. In addition to the above, caregivers should reside in the same home in Soweto as their family member diagnosed with dementia
4. Participants should be primarily responsible for activities of daily living for their family member, such as bathing, feeding, dressing, toileting, and medication management
5. Family caregivers should be taking care of their family member for at least 1 year
6. Caregivers should be comfortable conversing in English
7. Caregivers should be 18 years or older

Caregivers were contacted telephonically by the first author to invite them to participate in the research. During this process, caregivers were informed that their choice to participate in the study was voluntary and to clarify that there would be no costs to them. Participants were reimbursed for the travel costs incurred for transportation to the *Alzheimer’s South Africa* Soweto office, where interviews were scheduled. Written consent was obtained before data collection commenced. To ensure confidentiality and the protection of the participants’ identities, code names, such as ‘Family Caregiver 1’ (FC1), were used instead of using any real identifying data, and any identifying information was omitted. Sociodemographic information of participants is outlined in Table 2.

**Table 2.** Sociodemographic information of participants.

Sociodemographic Variable	Category	No of Participants
Age (years)	18–25	3
	25–40	5
	41–59	16
	60 +	6
Gender	Male	9
	Female	21
Religion	Christian	20
	Traditional african	4
	Spiritual	2
	No religion*	4
Employment status	Employed	3
	Unemployed	22
	Self-employed	3
	Pensioner	2
Highest level of education	Primary	1
	Secondary	18
	Tertiary	11
Type of housing	Formal (brick house)	25
	Informal settlement	5
Basic sanitation	Flush toilet inside the home	25
	Toilet outside the home	5
	
Water & electricity	Access	30
	No access	0
Transportation	Own car or family car	13
	Public transport	17

*Note: ‘No religion’ refers to the number of participants who stated that they do not ascribe to any religious denomination.

### Data collection

Data collection involved in-person, face-to-face interviews^2^ conducted by the first author using a semi-structured interview guide. Participants were asked open-ended questions to elicit their demographic characteristics such as age, level of education, employment, marital status and religious orientation. Thereafter, participants were asked to describe their area of residence, living conditions, any challenges they might have experienced and the resources available to them.

Prompting was used where necessary to elicit an in-depth understanding of caregiver needs. Each interview was approximately 60–90 min. Participants’ consent was obtained to audio record their interviews to allow for verbatim transcription. Due to time restrictions, interviews were transcribed by a transcription service to ensure the objectivity and accuracy of participants’ narratives.

## Data analysis

Data were analysed using Reflexive Thematic Analysis (RTA) outlined by Braun and Clarke (2006; 2019). This six-phase process entailed*: (1). Familiarisation with the data (2). Generating initial codes (3). Generating themes (4). Reviewing potential themes (5). Defining and naming themes 6). Producing the report.* Each process is briefly outlined in Table 3.

**Table 3.** Data analysis process.

Stage	Analysis process
Familiarisation with the data	The authors immersed themselves into the data by reading through the transcripts while simultaneously listening to the audio to ensure the accuracy of the transcriptions. This enabled the authors to recall the interview atmosphere and note any reflections or observations that emerged—each new reading and listening to the audio allowed for further insights.
Generating initial codes	Thorough notes were produced that reflected the transcripts and audio recordings. This created initial codes to organize the data into meaningful clusters based on the detail provided by the participant. Due to the copious amount of data generated, qualitative data analysis software – ATLAS.ti – was used to facilitate this step and assist with the subsequent thematic process.
Generating initial themes	Using ATLAS.ti, the connections between the identified codes were categorized into themes based on shared conceptualizations. Each category was assigned a descriptive label, and the meanings of and relationships between codes were deciphered.
Reviewing potential themes	Patterns across coded data were identified, and the entire data set was reviewed. Overlapping themes were collapsed and refined.
Defining and naming themes	A list of major themes and subthemes were categorised, and the narrative of the themes was identified and conceptualised within the broader story of the data set in response to the research questions.
Producing the report	A narrative account of participant data was presented using short excerpts from the transcripts to convey our salient findings in response to our research questions.

### Ensuring trustworthiness

This study used three methods to ensure the credibility of its findings: *Reflection,* where the author not only examines the thoughts of their research participants but is mindful of their own (Long & Johnson, 2000). A journal to enable author self-reflection was kept for the duration of the interview process. Information from all interviews and journal material was discussed between co-authors regularly. Thus, ensuring that any subjective biases that arise do not influence data interpretation. *Response validation* is vital to ensure that the collected information accurately reflects the intended message (Long & Johnson, 2000). The first author summarised her understanding of participants’ verbal narratives during the interview to validate their responses. This was to establish an accurate and clear understanding of the meaning of participants’ experiences. *Peer debriefing* occurs by verifying the data with colleagues on an ongoing basis to consider additional and alternative perspectives for the duration of the entire interview process (Long & Johnson, 2000). The authors discussed the interviews, themes and interpretations throughout the process.

## Findings

Four themes were generated through data analysis: (*1). Poverty, (2). Crime, Violence and Substance Abuse, (3). Practical Challenges and (4). A Sense of Normalcy*. Even though these themes have been categorized and presented independently, it should be emphasized that there are associative links between themes that contribute to each family caregiver’s experience within their context. Selected quotes with participant characteristics are included to illustrate the themes hereunder. A comprehensive overview of illustrative quotes is available as supplemental material.

### Poverty and unemployment

Most of the caregivers in this study disclosed being unemployed. They described experiencing multiple financial stressors, affecting their caregiving responsibilities, exacerbating their daily struggles within ‘township life’. Family caregivers expressed living ‘hand-to-mouth’ as they struggle to meet the daily needs of their households and their family members with dementia. Specifically, caregivers communicated that they do not have enough money to adequately cover their food, clothing, medical, transport and electricity. Some caregivers explained that ‘there’s no food’ or that their food supply was insufficient to sustain the household for the month (Q1-Q2)^3^, while others felt that they could not ‘maintain [the] standard’ that their family member was living due to the high costs associated with their dietary – ‘meat’, ‘fruits’, ‘fish’ and lifestyle – ‘vegan’ preferences (Q3–Q4). Other caregivers verbalized the strain of high expenses for clothing and incontinence products for their family members as their needs changed physically (Q5) or due to the change in season, describing this as ‘draining’ (Q6-Q7). In addition, high electricity costs were reported (Q7) – a caregiver explained that they had to use the fireplace instead of heaters to curb electricity expenses (Q8).

“Sometimes, there’s no food. I must make sure I must go and look for, lend the money to buy food, the money for pension I must pay that place, I pay R1200 and the other I must buy the food. So the food finish before month-end” (Q1 – 54 year old, female, self-employed)^4^

“What’s difficult is not having the finance to maintain that standard for her and basically leaving it up to people who don’t have those concerns and she’s now not in her full mind, has to sort of just take that, accept that. I mean, my mother was vegan, I think…so it’s hard watching her having to take what I know goes against her principles because of finance. It’s that, essentially” (Q3 – 40 years old, male, unemployed)

“And eventually as well, it’s costly, ja it is costly because the clothing, I had to buy lots of clothing, like I was saying she’s lost a lot of weight and you can’t be altering all those clothes, rather get her something. You know if you don’t have a support from your own siblings, it’s harder and I find put the strain to my children, though they’re helpful but sometimes you can’t be asking everything from them and asking for some help, I just wait for them to give whatever they maybe give and remembering I the only person, they have to do everything for themselves, it’s draining” (Q6 – 50 year old, female, unemployed)

“Food and electricity and the nappies, yeah its killing me” (Q7 – 74 year old, female, pensioner)

Moreover, caregivers expressed their struggle to access medical treatment or specific medications needed for their family members because ‘the money [is] finished’, ‘the doctor is expensive’ and ‘some of the medication is quite expensive’ at a pharmacy (Q9–Q11). Furthermore, the lack of funds for transportation was also mentioned by caregivers – albeit ‘mahala’, which is s Sotho word for ‘free’ healthcare at the clinic (Q12) – who emphasized the strain that this placed on them as an additional expense when medical care was needed (Q13). As a result, family members with dementia who required medical attention were unable to receive healthcare services (Q14).

“But now the challenge sometimes – oh, I wanted to say as well, I knew there was something I was forgetting – the medication. Some of her medications they refuse to pay for them, we have to pay cash, like this Donasep – it’s not on, she’s not able to take it on chronic. Everything we go to the chemist we need to pay for it. So some of the medication is quite expensive” (Q11 – 47 years old, female, employed)

“Because even though we can go in a Clinic mahala, but we needed transport to go there” (Q12 – 58 years old, male, employed)

“So I don’t have transport to take her back and she said to me I must, the doctor said I must bring her to, I must go for brain scan. So now I don’t have transport to take her to the hospital” (Q14 – 54 years old, female, unemployed)

Some caregivers directly attributed their financial struggles to a lack of education at school and their current unemployment status. A caregiver explicitly expressed her disappointment at the lack of ‘skills’ taught at schools that would have assisted with work opportunities to earn an income (Q15), while others alluded to the frustrations of being ‘poor’ (Q16) and the financial struggles of ‘not working’ (Q17) – which has hindered caregiver ability to provide for themselves and for their family members with dementia (Q18).

“I think with unemployment most of the thing…the problem we’re dealing with I think at schools they didn’t teach us what we should have been taught - maybe skills - they just teach us theories and stuff like that and have to see how you go on with life you see for people like us who didn’t go to varsity we don’t have any skills or anything we know nothing about work actually we just know nothing” (Q15 – 22 year old, male, unemployed)

“Basically it’s a loss of income, I think that’s my major challenge right now that I haven’t been able to take care of myself and give her the best care as well because of finances, so that has been my biggest struggle” (Q18 – 39 years old, female, unemployed)

### Crime, violence and substance abuse

The majority of caregivers highlighted the daily incidence of crime and violence in the township. Specifically, most caregivers described how burglaries occur within their neighbourhoods and lead to theft, rape and assault. This induced a sense of fear, worry and hypervigilance amongst family caregivers which necessitated precautionary measures, such as ‘hid[ing] the keys’ and ‘locking the gate all the time’ to keep their valuables and their family members with dementia safe (Q19–Q23). Of note, few caregivers disclosed being directly affected by criminal activities – burglaries and theft in particular, where their personal belongings were stolen from their homes (Q24–Q25). A caregiver further described the impact of this on their daily routine since their home was burgled multiple times and the challenge of getting her daughter safely to the bus stop while simultaneously managing the challenges of her husband’s dementia – related behaviour (Q26).

“You know the crime thing, ja the crime thing as we’re locking the gate all the time even if there is somebody at the house because you never know people they jump the fences…but one thing that you actually fear because sometimes they rape the grannies and you know that’s my fear” (Q20 – 40 year old, female, unemployed)

“Stealing at night…I remember one time I was renovating and then some of the things I had to take them out. Only to find out that in the morning my son was too lazy to put them back. The shoes, most of the things that he values, we did not even hear those people when they came in” (Q25 – 66 year old female, unemployed)

Furthermore, caregivers attributed criminal activity in the township to substance abuse (Q27). Most identified the use of *Nyaope*, a street drug, among the youth while others mentioned alcohol abuse via shebeens and taverns (Q28) in some areas that resulted in theft, which threatened the safety of the township (Q29). According to caregivers, ‘kids’ who use *Nyaope* ‘are marking people’ (Q30), ‘get[ting] into a person’s house [and] steal[ing]’ (Q31) and others using the same drug ‘will end up doing crime because they need to get a fix’ (Q32).

“Because like recently we see that now there is a lot of like youth they are on drugs and there is a group of young children you know, taking drugs and whenever they are on drugs, they are marking people, they are stealing and fortunately in my house they did not enter but in some other houses they did enter. Stole TV, you know, electricity appliances” (Q30 – 41 years old, female, unemployed)

Notably, a caregiver verbalized her proactive attempts to keep her community safe by arranging for ‘patrollers’ to be paid to protect people from ‘those tsotsi’s’ who ‘[search]’, ‘[stab]’ and ‘shoot’ (Q33).

“…and they want to get knife, they are searching people, stabbing people, they shoot people – those tsotsi’s. Ya, I organise those patrollers. But then they are sleeping at night. So each and every house must donate R30.00 so we must pay those patrollers” (Q33 – 54 years old, female, self-employed )

### Practical challenges

Most caregivers identified practical challenges in their home that contribute to their daily struggles as dementia caregivers. Caregivers explained that the toilet inside their home is too far away for their family member with dementia to access (Q34), there are not enough beds in the home for everyone to sleep on, as the family member with dementia uses the ‘whole bed’, and the family ‘[doesn’t] have much’ (Q35), there is no geyser in the home, so the family has to heat water in a kettle first in order to bath their family member in warm water (Q36). Of note, a common practical challenge among caregivers was a lack of space inside or outside their homes for a bathroom, so caregivers had to either use a plastic dish (Q37) or a bucket to bath or to use as a toilet (Q38) for their family member with dementia. Similarly, for caregivers who did have a ‘normal bathroom’ inside their homes, space was still a challenge as it was insufficient to push a wheelchair through the home to facilitate easier mobility for the caregiver and their family member with dementia (Q39).

“You see my mother is using the dish, to bath, to wash. It’s a big plastic. There’s no space for bathroom because the yard is too small” (Q37 – 54 years old female, self-employed)

“Like the bathroom now is the normal bathroom, it’s inside the house. But if it was bigger, then you can actually put something to fit her so that when she washes, you know, like she can slide in with the wheelchair. So, definitely challenges like the rooms are not big enough and the bathroom is not big enough” (Q39 – 50 years old, female, unemployed)

Caregivers who mentioned being able to make home modifications to suit the practical needs of their family member with dementia were a minority – a family was able to ‘build ramps’, install a bathroom rail, and change the light fittings for their family member’s convenience, safety and to facilitate her independence (Q40). Another family was able to ‘build rails’ in their home for their family member’s safety (Q41).

“We build ramps, we changed our toilets. We put a rail so that she can hold on to it in the toilet. So she does not need help in there, she just does it herself. Lighting also, we needed to change the lights in her bedroom to make it brighter. And also my dad got her these lights where you press on a remote, instead of going to a wall, so that if she is in bed and she thinks she sees something she can just switch on the light and see oh there is nothing there” (Q40 – 30 years old, female, unemployed)

“Ja so it meant that I had to buy material now to actually build rails, for her own safety” (Q41 – 39 years old, female, unemployed)

### A sense of normalcy

When caregivers were asked if the area in Soweto that they lived in was safe, most caregivers responded positively and stated that it was. However, there was a prominent sense of normalcy – even among caregivers who did not perceive their area as safe. As caregivers reflected on this, they expressed the notion ‘I grew up here, I do not know the difference of safe and not safe…it is normal to me’ (Q42). Others highlighted that their own street might be safe, but other areas in the township known as ‘zones’ might ‘have their problems’ due to ‘different languages’ (Q43). Furthermore, caregivers who reported on crime and violence in the township reflected on these incidences as ‘nothing’ and stated ‘it’s not safe, but it’s okay because we are aware of the things that can put you in trouble’ (Q44). Of note, there appeared to be a hesitance to ‘complain’ (Q45) about the challenges in the township as these caregivers believed ‘you’ll just deal with it when it happens to you’ (Q46).

“For everyone, the perception with people from outside Soweto is that Soweto is not safe. So, I grew up here, I do not know the difference of safe and not safe. Maybe if I stayed in the suburb for five years but to find people in the suburbs who are complaining about crime and all that so, I do not know how to say safe but it is normal to me” (Q42 – 51 years old male, unemployed)

“Things like the other day some people got shot and they were being robbed in their own property. They gave people their things. Like people came and they wanted things maybe TV's and stuff. Then they say they will co-operating but they got shot. But it’s nothing, it's been happening in Soweto. So it's nothing you get scared or you'll just deal with it when it happens to you” (Q46 – 28 years old, male, employed)

## Discussion

The findings of this study depict the socioeconomic conditions of family caregivers living in Soweto, the largest township in South Africa, and its impact on dementia caregiving. Majority of the family caregivers in this study were unemployed and identified the financial aspects of caregiving as a major strain, as they struggled to meet the needs of their family members with dementia over and above their usual expenses. Due to already ‘poor’ living conditions, where caregivers were living ‘hand-to-mouth’, financial resources were inept to fully cater for food, clothing, incontinence products, medication and transport costs needed to sustain the well-being of their family member with dementia. The high costs of caregiving is a taxing, shared struggle consistent across South Africa (Bosch, 2014; Gurayah, 2015; Hendriks-Lalla & Pretorius, 2018; Valoo, 2016) and international samples (Ainamani et al., 2020; Lai, 2012; Lindeza et al., 2020; Owokuhaisa et al., 2020; Roberts & Struckmeyer, 2018; Stall et al., 2019) regardless of socioeconomic circumstances. However, as demonstrated in this study, financial struggles exacerbate caregiver burden particularly in rural areas and townships where poverty and unemployment persist (Ainamani et al., 2020; Bosch, 2014; Gurayah, 2015; Hendriks-Lalla & Pretorius, 2018) and family caregivers are not financially compensated for their caregiving tasks (Bosch, 2014; Guerchet et al, 2017; Kehoua et al., 2019). As a consequence, some family caregivers were unable to access medical care or healthcare services for their family members in this study, as the need for food had to be prioritized over transportation costs. Decisions such as these, together with the financial struggles that caregivers experienced as described above, significantly contributed to the frustrations that family caregivers expressed.

Additionally, caregivers in this study felt fearful, worried and concerned for the safety of their family members due to the high prevalence of crime, violence and substance abuse in the township. Caregivers specified criminal and violent activities such as theft, rape and assault that occurred frequently in the township and their protective behaviours such as keeping their homes locked at all times and hiding their keys. According to caregivers, this was to prevent their family members from wandering outside, where they may encounter a dangerous situation or allowing burglars to enter their homes. Of note, there were no reports of direct harm inflicted by criminal activities onto any family member with dementia in this study. Furthermore, caregivers also highlighted the link between criminal activities and the substance abuse problem in the township. They predominantly referred to *Nyaope*, a street drug in South Africa, which is comprised of a blend of cannabis, heroin and antiretroviral (ARV) medication (Mthembi et al., 2019). Research conducted regarding people with dementia safety in communities have reported on caregiver attempts to prevent ‘physical, economic, emotional and relational harm’ (Häikiö et al., 2019, p.9) as a result of patient behaviours and issues with healthcare services (Behrman et al., 2017). Contextual or socioeconomic variables that may impact caregiver safety have not been previously reported on as in this study.

Similarly, practical challenges that may affect dementia caregiving have also been scarce in the literature. In this study, family caregivers stated the difficulties that they encountered in their homes that directly impacted caregiving tasks. Specifically, caregivers reported constraints in terms of the resources and infrastructure in their homes needed to carry out caregiver tasks, which residents of South African townships have to endure (Pretorius & Theron, 2018; Theron et al., 2021). This included insufficient bathroom essentials such as a geyser to access warm water or enough beds to sleep on for all residents in the home. In addition, severe spatial restrictions (Nadat & Jacobs, 2021; Pretorius & Theron, 2018) were also highlighted by some caregivers which did not allow for a bathroom or toilet inside or outside the home that was conducive to the family member with dementia’s needs. Hence, caregivers explained that they had to use a ‘plastic dish’ or a ‘bucket’ to bath their family member. Furthermore, the spatial organization inside the home of some caregivers did not allow for a wheelchair or other modifications to be made for those who could afford it. Only two caregivers in this study were able to make some changes in their homes to accommodate their family members with dementia’s needs.

In general, there was a sense of normalcy that caregivers alluded to – whether or not they perceived their township as safe and a hesitance to identify practical challenges that they might be facing. As caregivers reflected, *‘It’s like township life, everyone accepts it.’* This sentiment implies that ‘disadvantage and suffering are the norm… where adversity is more like a common fate’ in contextually disadvantaged and under-resourced communities (Theron, 2015, p. 25). Of significance, it appears that despite the risk factors such as poverty, unemployment, violence, crime and material challenges within the lived context of the caregivers in this study, and among many Black South Africans (Theron & Ungar, 2019) their positive responses and adaptability to these adverse circumstances indicate resilience (Masten, 2018; Mosavel et al., 2015). Moreover, studies have documented that providing more care, over a lengthy period of time, accessing support services and caring for a female, was associated with high resilience among dementia caregivers (Gaugler et al., 2007; Joling et al., 2016).

## Limitations

The qualitative design of our study could be considered a limitation. Caregivers in this study appeared guarded when discussions on contextual factors and challenges occurred. Perhaps a mixed-methods approach with the inclusion of a quantitative component would have been more appropriate to draw measurable and objective conclusions regarding the contextual factors in the township that may affect the dementia family caregiver experience. This would have allowed for a more holistic, rigorous and structured examination of the sociodemographic variables and its correlations to dementia family caregiving. Future studies should be conducted using a mixed-methods approach.

## Conclusion and recommendations

This study forms part of a larger study to explore experiences of dementia family caregivers in a Black African township in South Africa. The focus of this paper was to elucidate the sociodemographic factors in the township that may affect the dementia family caregiver experience. Our findings were consistent with the large body of research that highlight the financial stressors that contribute to dementia caregiver burden (Ainamani et al., 2020; Bosch, 2014; Gurayah, 2015; Hendriks-Lalla & Pretorius, 2018; Lai, 2012; Lindeza et al., 2020; Owokuhaisa et al., 2020; Roberts & Struckmeyer, 2018; Stall et al., 2019; Valoo, 2016). However, this study links a contextual layer as a nuance to the family caregiver experience by characterizing the impact of socioeconomic conditions such as poverty, unemployment, crime, violence and substance abuse. These conditions not only exacerbated the financial burden for family caregivers, as evidenced by their impoverished living conditions and lack of financial resources and stability, but also contributed to the psychological distress emphasized in the larger study. Family caregivers in this study were concerned and fearful for the safety of their family members due to the dangers that this socioeconomic context posed. Although few caregiver households were victims of burglaries, none of the caregivers in this study reported on harm directly inflicted onto their family members with dementia.

Another nuanced layer that this study provides is the practical challenges that some family caregivers reported such as spatial constraints and insufficient material resources such as bathroom essentials – structural disadvantages and deprivation that are characteristic of South African townships. As a consequence, these caregivers were unable to make necessary adjustments in their homes to meet their family members with dementia’s needs. Of significance, caregivers in this study tentatively described practical challenges that they endured and adopted a permissive stance – maintaining a sense of normalcy and adaptation despite the adverse conditions in which they live.

In light of these findings, further research should be conducted to strengthen our efforts to effect change and to advocate for the safety and resource needs of dementia caregivers. We propose the focus on quantitative research on a large scale that includes epidemiological (De Jager et al., 2017) and cost studies to inform policy making and resource allocation for under-resourced communities (Vandepitte et al., 2020).

Furthermore, it is imperative that community advocacy groups and non-governmental organizations are mobilized to create social and financial support programmes in townships to reduce the psychosocial and financial burden that family caregivers endure. Similarly, government organizations need to be engaged with to promote public health policies, feeding schemes, financial aid and security on a larger scale nationally to specifically address the needs of people with dementia and their caregivers.

## Supplemental Material

Supplemental Material - Exploring the contextual factors that impact the dementia family caregiving experience in Soweto township, South Africa Click here for additional data file. Supplementary Material for Exploring the contextual factors that impact the dementia family caregiving experience in Soweto township, South Africa by Aqeela Mahomed and Chrisma Pretorius in Dementia

## Notes

1. Black, White, Coloured and Indian are the predominant racial categories used to classify different population groups in South Africa imposed by the Apartheid regime.

2. Data collection occurred during 2019, before the emergence of the COVID-19 pandemic.

3. Quote numbers 1–46 correspond to participant quotes provided as supplemental material.

4. Refer to supplemental material for further detailed illustrative quotes.
