# Supplementary material for: Exploring the contextual factors that impact the dementia family caregiving experience in Soweto township, South Africa
Source: Dementia (London). 2022 Aug 8;21(7):2231–47. doi: 10.1177/14713012221117905 (PMC9483691; doi:10.1177/14713012221117905)
Supplement: Supplemental Material - Exploring the contextual factors that impact the dementia family caregiving experience in Soweto township, South Africa [file sj-pdf-1-dem-10.1177_14713012221117905.pdf]

Supplemental Material: Semi-Structured Interview Guide

1. I am interested to know more about you – can you tell me more about yourself? *(Prompts – age, level of education, employment and marital status etc.)*
2. Please describe the place / area that you and your family member with dementia live in. *(Prompts – access to basic sanitation, water, electricity, assets in the home)*
3. What are the challenges that you are dealing with in your home and in the township?
4. What makes it difficult to take care of your family member with dementia?
5. Please tell me about the things that you have to take care of your family member with dementia.
6. Please tell me about the things that you don't have to take care of your family member with dementia.

Supplemental Material: Comprehensive overview of themes and illustrative quotes

**Themes**	**Illustrative Quotes**
**Theme 1: Poverty**	"Sometimes there's no food. I must make sure, I must go and look for, lend the money to buy food, the money for pension I must pay that place, I pay 1.2 and the other I must buy the food. So the food finish before month-end" (Q1)<br>"Two weeks of the month I can do, but the two weeks … but the last week of the month everything is finish. You see. Even the food is finish. So when it come in that situation, yeah I'm short…" (Q2)<br>"What's difficult is not having the finance to maintain that standard for her and basically leaving it up to people who don't have those concerns and she's now not in her full mind, has to sort of just take that, accept that. I mean my mother was vegan I think…so it's hard watching her having to take what I know goes against her principles because of finance. It's that, essentially" (Q3)<br>"Because she eats a lot, she wants food and she likes meat, and meat is expensive. So when you give her beans and some things, sometimes you can just see the face, so yeah, and you must have fruits, you must change her diet – you can't give her one thing all the time. So that becomes expensive because you must make sure – you can't give her pap all the time, you can't give her bread all the time. You have to change and give a range of things so that she can be able to eat, but she likes meat. Likes meat and likes fish" (Q4)<br>"Yes diapers have become very expensive because I can't use these wrap-around ones, I use the pull-ups, full ones, they are convenient for me. Because just once he must hold on the side when you press this side and he can't do things for himself, so diapers, very heavy" (Q5)

	“And eventually as well, it’s costly, ja it is costly because the clothing, I had to buy lots of clothing, like I was saying she’s lost a lot of weight and you can’t be altering all those clothes, rather get her something. You know if you don’t have a support from your own siblings, it’s harder and I find put the strain to my children, though they’re helpful but sometimes you can’t be asking everything from them and asking for some help, I just wait for them to give whatever they maybe give and remembering I the only person, they have to do everything for themselves, it’s draining” (Q6)<br>“Food and electricity and the nappies, yeah its killing me” (Q7)<br>“But this year I had to use the fireplace because I’ve got a sick person and I can’t use heaters. Electricity was killing us” (Q8)<br>“Yeah. Sometimes she's sick. Sometimes I think I want to take her to go there and … because money it's finished now I can't take there” (Q9)<br>“I don’t have money for a doctor for the clinic to hospital. The doctor is expensive. R300 and something” (Q10)<br>“But now the challenge sometimes – oh, I wanted to say as well, I knew there was something I was forgetting – the medication. Some of her medications they refuse to pay for them, we have to pay cash, like this Donasep – it’s not on, she’s not able to take it on chronic. Everything we go to the chemist we need to pay for it. So some of the medication is quite expensive” (Q11)<br>“Because even though we can go in a Clinic mahala, but we needed transport to go there” (Q12)<br>“They say must take to the doctor, they say they don’t have money to take her, because the money – they’re supposed to eat” (Q13)<br>“So I don’t have transport to take her back and she said to me I must, the doctor said I must bring her to, I must go for brain scan. So now I don’t have transport to take her to the hospital” (Q14)<br>“I think with unemployment most of the thing…the problem we’re dealing with I think at schools they didn’t teach us what we should have been taught - maybe skills - they just teach us theories and stuff like that and have to see how you go on with life you see for people like us who didn’t go to varsity we don’t have any skills or anything we know nothing about work actually we just know nothing” (Q15)<br>“Ja and people who are poor and unemployed yes. It is too much” (Q16)<br>“As a caregiver as I am not working, to be honest, I really need a job because financially I am struggling. I am really struggling financially” (Q17)<br>“Basically it’s a loss of income, I think that’s my major challenge right now that I haven’t been able to take care of myself and give her the best care as well because of finances, so that has been my biggest struggle” (Q18)
**Theme 2: Crime, Violence and Substance Abuse**	“We live in Soweto so, there’s gunshots, there’s all kind of things happening and she wants to get out, so you have to do whatever you can, so if I had to hide the keys, rather she fights break things in the house, it’s fine…it’s more dangerous if she goes out in the street” (Q19)<br>“You know the crime thing, ja the crime thing as we’re locking the gate all the time even if there is somebody at the house because you never know people they jump the fences…but one thing that you actually fear because sometimes they rape the grannies and you know that’s my fear” (Q20)<br>“There is the high crime rate… it is a quiet place, and then she forgets, sometimes she forgets to lock the gate and then she will go to that house and she stay there about two hours or four hours and then sometimes I come from work, the gates are open and the doors are open. These boys they are looking at you. Then they disappear, some of the stuff is gone, the TV is gone, the microwave is gone” (Q21)<br>“Ja, that is why I lock the house because like you will never know, maybe someone is standing there. She, he sees that you are going out and my mother and this son of mine they are going outside. He will just come in and take something, you see? Ja, that is why I lock the house when I go” (Q22)

	"Her safety. I am worried about her safety" (Q23)<br>"My room was broken into, and they stole my things, a pair of sneakers and sound and computer and stuff, ja" (Q24)<br>"Stealing at night…I remember one time I was renovating and then some of the things I had to take them out. Only to find out that in the morning my son was too lazy to put them back. The shoes, most of the things that he values, we did not even hear those people when they came in" (Q25)<br>"The challenges are there is too much break ins, in the past we had three break ins and in the morning, in the morning when people go to work, some people who leave very early and I've been robbed along the way, so before my daughter got the transport that takes them to work, every morning we used to accompany her to the bus stop every morning, I would go with her or her father and now ever since he's the way he is, sometimes he wants to wake up, sometimes he doesn't want to wake up and when I'm out, taking her to the bus stop, he wants to come, he will wake up and go sonder skoene [without shoes] and follow us, he doesn't never lock the house, so I must be taking the keys when there are days and then and now that there's a transport for her, we don't go out but it's not safe and in the evening, you must always have somebody to wait for you when you get off the taxi" (Q26)<br>"The theft around the house, stealing of copper cables, and that … all that kind of things, smaller things…there's these kids that take drugs…yes, so they take these kinds of things, steal whatever, they go and sell them so they can get money to buy the drug, so that's a challenge" (Q27)<br>"We're in the centre of taverns all around us. So where there are a lot of taverns and drunk people walking around at night, obviously, it's going to be a good spot for people who rob people to stand there at night, wait for people coming back home or going to the tavern" (Q28)<br>"Drugs had made Soweto not to be a safe place no more. You know. Because they would do anything for those things" (Q29)<br>"Because like recently we see that now there is a lot of like youth they are on drugs and there is a group of young children you know, taking drugs and whenever they are on drugs, they are marking people, they are stealing and fortunately in my house they did not enter but in some other houses they did enter. Stole TV, you know, electricity appliances" (Q30)<br>"I do not [know] whether you know these kids that's smoking drugs, Nyaope, they steal from other people's houses. They get into a person's house - they steal" (Q31)<br>"There is drug problem and theft. Those are the kind of issues that we have to deal with because people who are taking nyaope for them to get fixed they will do anything…they will end up doing crime because of they need to get a fix" (Q32)<br>"…and they want to get knife, they are searching people, stabbing people, they shoot people – those tsotsi's. Ya, I organise those patrollers. But then they are sleeping at night. So each and every house must donate R30.00 so we must pay those patrollers" (Q33)
**Theme 3: Practical Challenges**	"There is a toilet in the house but it's quite far the toilet is on the other side by this long passage if anyone wants to go to the bathroom" (Q34)<br>"We don't have much. Like we haven't done any changes yeah. She sleeps at the whole bed and we only have couches. You know. But we haven't … we don't have much" (Q35)<br>"Oh and then the thing is that right now we don't have what you call, a geyser. Yes that is the problem, because when she wash we need to put water in with a kettle and put it in the bucket so that we can put it in the bath" (Q36)<br>"You see my mother is using the dish, to bath, to wash. It's a big plastic. There's no space for bathroom because the yard is too small" (Q37)<br>"She must use the bucket in the morning, must take it to the toilet in the bathroom, empty it and put the Domestos inside because it can smell. Then it stands outside, we take it in in the afternoon before we sleep" (Q38)

	“Like the bathroom now is the normal bathroom, it’s inside the house. But if it was bigger, then you can actually put something to fit her so that when she washes, you know, like she can slide in with the wheelchair. So, definitely challenges like the rooms are not big enough and the bathroom is not big enough” (Q39)<br>“We build ramps, we changed our toilets. We put a rail so that she can hold on to it in the toilet. So she does not need help in there, she just does it herself. Lighting also, we needed to change the lights in her bedroom to make it brighter. And also my dad got her these lights where you press on a remote, instead of going to a wall, so that if she is in bed and she thinks she sees something she can just switch on the light and see oh there is nothing there” (Q40)<br>“Ja so it meant that I had to buy material now to actually build rails, for her own safety” (Q41)
**Theme 4: A Sense of Normalcy**	“For everyone, the perception with people from outside Soweto is that Soweto is not safe. So, I grew up here, I do not know the difference of safe and not safe. Maybe if I stayed in the suburb for five years but to find people in the suburbs who are complaining about crime and all that so, I do not know how to say safe but it is normal to me” (Q42)<br>“Well it is safe, let’s say the street that I live in… the street is safe on its own but then the zone have their problems we cannot say it’s safe because you’ve got different languages” (Q43)<br>“…and robberies, they mug you when you come from somewhere where it’s dark and so forth, but has subsided. It is not safe, but it’s okay because we are aware of the things that can put you in trouble” (Q44)<br>“Ja I’m not complaining, there are, you do have, a break in now and again but we haven’t had any problems” (Q45)<br>“Things like the other day some people got shot and they were being robbed in their own property. They gave people their things. Like people came and they wanted things maybe TV's and stuff. Then they say they will co-operating but they got shot. But it's nothing, it's been happening in Soweto. So it's nothing you get scared or you'll just deal with it when it happens to you” (Q46)
